# Addendum: The observation of vibrating pear-shapes in radon nuclei

**DOI:** 10.1038/s41467-020-17309-y

**Published:** 2020-07-13

**Authors:** P. A. Butler, L. P. Gaffney, P. Spagnoletti, J. Konki, M. Scheck, J. F. Smith, K. Abrahams, M. Bowry, J. Cederkäll, T. Chupp, G. de Angelis, H. De Witte, P. E. Garrett, A. Goldkuhle, C. Henrich, A. Illana, K. Johnston, D. T. Joss, J. M. Keatings, N. A. Kelly, M. Komorowska, T. Kröll, M. Lozano, B. S. Nara Singh, D. O’Donnell, J. Ojala, R. D. Page, L. G. Pedersen, C. Raison, P. Reiter, J. A. Rodriguez, D. Rosiak, S. Rothe, T. M. Shneidman, B. Siebeck, M. Seidlitz, J. Sinclair, M. Stryjczyk, P. Van Duppen, S. Vinals, V. Virtanen, N. Warr, K. Wrzosek-Lipska, M. Zielinska

**Affiliations:** 1 Oliver Lodge Laboratory, University of Liverpool, Liverpool, L69 7ZE UK; 2 CERN, Geneva, 23 CH-1211 Switzerland; 3 School of Computing, Engineering and Physical Sciences, University of the West of Scotland, Paisley, PA1 2BE UK; 4 Department of Physics & Astronomy, University of the Western Cape, Private Bag X17, Bellville, 7535 South Africa; 5 TRIUMF, Vancouver, V6T 2A3 BC Canada; 6 Physics Department, Lund University, Box 118, Lund, SE-221 00 Sweden; 7 Department of Physics, University of Michigan, Ann Arbor, 48104 MI USA; 8 INFN Laboratori Nazionali di Legnaro, Legnaro, 35020 PD Italy; 9 Instituut voor Kern- en Stralingsfysica, KU Leuven, Leuven, B-3001 Belgium; 10 Department of Physics, University of Guelph, Guelph, N1G 2W1 Ontario Canada; 11 Institute for Nuclear Physics, University of Cologne, Cologne, 50937 Germany; 12 Institut für Kernphysik, Technische Universität Darmstadt, Darmstadt, 64289 Germany; 13 Heavy Ion Laboratory, University of Warsaw, Warsaw, PL-02-093 Poland; 14 Department of Physics, University of Jyvaskyla, P.O. Box 35, Jyvaskyla, FIN-40014 Finland; 15 Helsinki Institute of Physics, P.O. Box 64, Helsinki, FIN-00014 Finland; 16 Department of Physics, University of Oslo, P.O. Box 1048, Oslo, N-0316 Norway; 17 Department of Physics, University of York, York, YO10 5DD UK; 18 JINR Dubna, Dubna, 141980 Moscow Region Russia; 19 Consejo Superior De Investigaciones Científicas, Madrid, S 28040 Spain; 20 IRFU CEA, Université Paris-Saclay, Gif-sur-Yvette, F-91191 France

**Keywords:** Exotic atoms and molecules, Experimental nuclear physics

Addendum to: *Nature Communications* 10.1038/s41467-019-10494-5, published online 6 June 2019.

We would like to make readers aware that after the publication of this article the sort code was updated. This resulted in more gamma–gamma data, particularly for high-spin transitions. By performing additional analysis we confirm the energies of most of the states, have identified several new states, and have updated one of the states that was incorrectly represented in its energy in the original paper. The figures (Figs. 1–4) and table (Table 1) are updated along with their captions. The overall conclusions of the paper remain unaffected.

The new data confirm our original finding, that ^224,226^Rn behave as octupole vibrators in which the octupole phonon is aligned to the rotational axis. We conclude that there are no isotopes of radon that have static octupole deformation, so that any parity doublets in the odd-mass neighbours will not be closely spaced in energy. This means that radon atoms will provide less favourable conditions for the enhancement of a measurable atomic electric-dipole moment.

Prior to this work, less was known about the energies and spins of excited states in ^224,226^Rn. The spectra of γ-rays time-correlated with scattered beam and target recoils are shown in Fig. 1. The *E*2 γ-ray transitions within the ground-state positive-parity band can be clearly identified. The other relatively intense γ-rays observed in these spectra are assumed to have *E*1 multipolarity, depopulating the odd-spin negative-parity members of the octupole band. In order to determine which states are connected by these transitions, pairs of time-correlated (‘coincident’) γ-rays were examined. In this analysis, the energy spectrum of γ-rays coincident with one particular transition is generated by requiring that the energy of this gating transition lies in a specific range. Typical spectra obtained this way are shown in Fig. 2. Each spectrum corresponds to a particular gating transition, background subtracted, so that the peaks observed in the spectrum arise from γ-ray transitions in coincidence with that transition.

The data in these γ–γ spectra have been significantly enhanced by modifying the sort code that converts raw data from the Miniball spectrometer to Root analysis files. This modification enables γ–γ data to be included when both heavy ions are detected in the silicon detector array, in addition to single-ion events. Since both conditions for ion detection were already considered for single γ-ray events, this modification does not affect the total spectra shown in (Fig. 1), but considerably enhances the statistics of the γ–γ gated spectra shown in Fig. 2. The additional γ–γ data have allowed the authors to extend the level schemes by one additional state in each of the positive-parity bands in ^224,226^Rn and in the negative-parity band in ^224^Rn over that reported originally. More importantly, we are able to determine the probable energy of the 7^−^, 9^−^ and 11^−^ states in ^226^Rn, see Fig. 2. By extrapolating this band to lower spin states on the basis of its rotational-like behaviour, we are able to estimate the energies of the 5^−^ and 3^−^ states, whose decays to the positive-parity band are observed in the total γ-ray spectrum, see Fig. 1. These tentative assignments imply that the energy of the $5^ - \to 4^ +$ transition is 565 keV, not 585 keV as reported originally. The gated coincidence spectra for both candidate $5^ - \to 4^ +$ transitions are shown in Fig. 2. In both cases the quality of the gated spectra does not allow a firm assignment to be made to the γ-ray transition. The spectrum gated by the candidate $3^ - \to 2^ +$transition is featureless, as expected given the large internal conversion of the $2^ + \to 0^ +$ transition. The Doppler-corrected energies for transitions in ^224^Rn and ^226^Rn together with the deduced level energies are given in Table 1. The newly extended level schemes for ^224,226^Rn constructed from the coincidence spectra, together with that of ^222^Rn, are shown in Fig. 3.

From the new level schemes, we are able to extend the knowledge of the systematics of the low-lying energy levels and difference in aligned angular momentum, $\Delta i_x = i_x^ - - i_x^ +$ as a function of rotational frequency *ω*, see Fig. 4. For octupole vibrational nuclei in which the negative-parity states arise from coupling an octupole phonon to the positive-parity states, it is expected that $\Delta i_x\sim 3\hbar$ as the phonon prefers to align with the rotational axis. This is the case for the isotopes ^218,220,222,224,226^Rn. The new measurements reinforce the original conclusion, that, if measurable CP-violating effects occur in nuclei, the enhancement of nuclear Schiff moments arising from octupole effects in odd-*A* radon nuclei is likely to be much smaller than for heavier octupole-deformed systems.

**Fig. 1:**
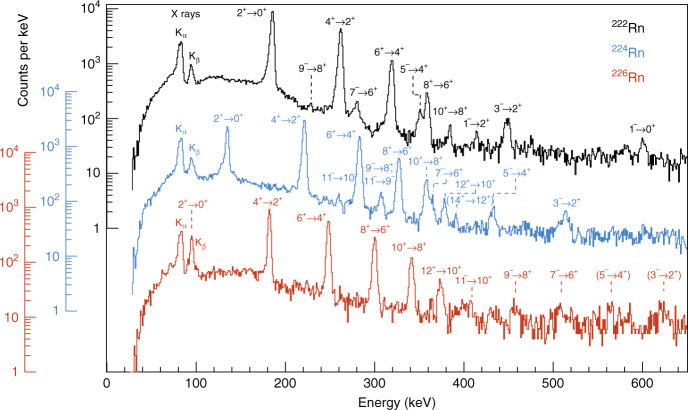
Spectra of γ-rays. The γ-rays were emitted following the bombardment of ^120^Sn targets by ^222^Rn (black), ^224^Rn (blue) and ^226^Rn (red). The γ-rays were corrected for Doppler shift assuming that they are emitted from the scattered projectile. Random coincidences between Miniball γ-ray and silicon particle detectors have been subtracted. The transitions which give rise to the observed full-energy peaks are labelled by the spin and parity of the initial and final quantum states. The assignments of the transitions from the negative-parity states in ^224,226^Rn are tentative (see text).

**Fig. 2:**
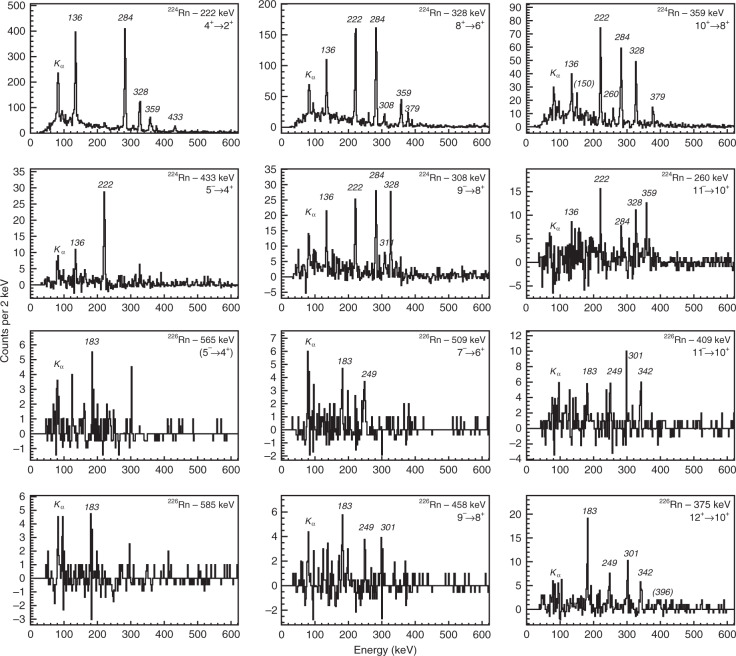
Coincidence γ-ray spectra. The representative background-subtracted γ-ray spectra are in time-coincidence with different gating transitions. Here the observed peaks are labelled by the energy (in keV) of the transition. The gating transition is additionally labelled by the proposed spin and parity of the initial and final states.

**Fig. 3:**
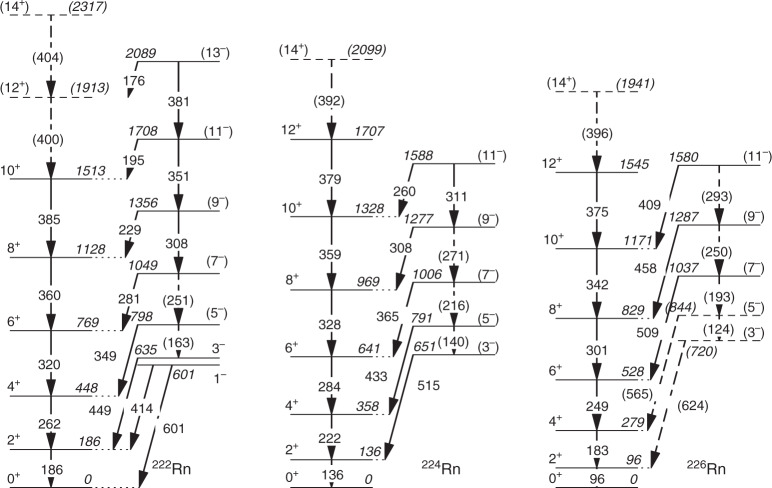
Level schemes. These partial level-schemes for ^222,224,226^Rn show the excited states of interest. Arrows indicate γ-ray transitions. All energies are in keV. Firm assignments of transitions and energy levels in the scheme are from previous work or have been made using γ–γ-coincidence relations; tentatively assigned transitions such as those from the 3^−^ and 5^−^ states in ^226^Rn are shown as dashed lines. The assignment of spins and parities in brackets are tentative.

**Fig. 4:**
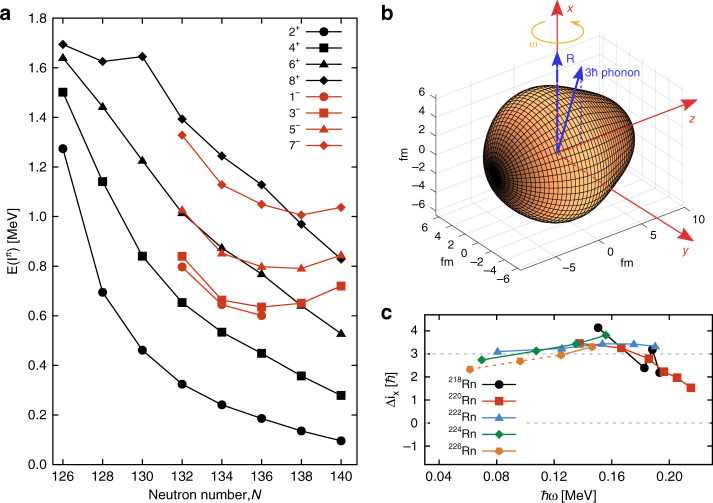
Systematic behaviour of radon isotopes. **a** Systematics of the energies for different spins of low-lying positive-parity (black) and negative-parity states (red) in radon isotopes; **b** cartoon illustrating how the octupole phonon vector aligns with the rotation (*R*) vector (which is orthogonal to the rotating bodyʼs symmetry axis) so that *I* = *R* + 3$\hbar$ and $\Delta i_x = 3\hbar$; **c** difference in aligned spin for negative- and positive-parity states in ^218-224^Rn. The dashed line at $\Delta i_x = 0$ is the expected value for static octupole deformation.

**Table 1:** Energies of levels and transitions in ^224^Rn and ^226^Rn.

^224^Rn *E*_level_ (keV)	$I_i^\pi$	*E*_γ_ (keV)	$I_f^\pi$
135.6 (5)	2^+^	135.6 (5)	0^+^
357.6 (6)	4^+^	222.0 (5)	2^+^
641.4 (8)	6^+^	283.8 (5)	4^+^
969.2 (9)	8^+^	327.8 (5)	6^+^
1327.8 (10)	10^+^	358.6 (5)	8^+^
1706.8 (11)	12^+^	379.1 (5)	10^+^
(2098.7) (13)	(14^+^)	(391.8) (6)	12+
650.6 (8)	(3^−^)	515.0 (6)	2^+^
790.8 (8)	(5^−^)	433.2 (5)	4^+^
1006.4 (10)	(7^−^)	365.0 (5)	6^+^
1277.2 (10)	(9^−^)	308.0 (5)	8^+^
1588.3 (13)	(11^-^)	260.5 (8)	10^+^

^226^Rn *E*_level_ (keV)	$I_i^\pi$	*E*_γ_ (keV)	$I_f^\pi$
96.0 (11)	2^+^	96.0 (11)	0^+^
278.9 (12)	4^+^	182.9 (5)	2^+^
527.9 (13)	6^+^	249.0 (5)	4^+^
828.6 (14)	8^+^	300.7 (5)	6^+^
1170.8 (14)	10^+^	342.1 (5)	8^+^
1545.4 (15)	12^+^	374.6 (5)	10^+^
(1941.2) (19)	(14^+^)	(395.8) (11)	12^+^
(719.9) (17)	(3^−^)	(623.9) (13)	2^+^
(843.6) (14)	(5^−^)	(564.7) (8)	4^+^
1036.8 (16)	(7^−^)	508.9 (9)	6^+^
1286.7 (17)	(9^−^)	458.1 (10)	8^+^
1579.6 (18)	(11^−^)	408.8 (11)	10^+^

The 1-σ errors are given, estimated from the statistical error and the uncertainty in the energy calibration and Doppler correction.

