# Publisher Correction: The observation of vibrating pear-shapes in radon nuclei

**DOI:** 10.1038/s41467-020-19081-5

**Published:** 2020-10-09

**Authors:** P. A. Butler, L. P. Gaffney, P. Spagnoletti, J. Konki, M. Scheck, J. F. Smith, K. Abrahams, M. Bowry, J. Cederkäll, T. Chupp, G. de Angelis, H. De Witte, P. E. Garrett, A. Goldkuhle, C. Henrich, A. Illana, K. Johnston, D. T. Joss, J. M. Keatings, N. A. Kelly, M. Komorowska, T. Kröll, M. Lozano, B. S. Nara Singh, D. O’Donnell, J. Ojala, R. D. Page, L. G. Pedersen, C. Raison, P. Reiter, J. A. Rodriguez, D. Rosiak, S. Rothe, T. M. Shneidman, B. Siebeck, M. Seidlitz, J. Sinclair, M. Stryjczyk, P. Van Duppen, S. Vinals, V. Virtanen, N. Warr, K. Wrzosek-Lipska, M. Zielinska

**Affiliations:** 1 Oliver Lodge Laboratory, University of Liverpool, Liverpool, L69 7ZE UK; 2 CERN, Geneva, 23 CH-1211 Switzerland; 3 School of Computing, Engineering and Physical Sciences, University of the West of Scotland, Paisley, PA1 2BE UK; 4 Department of Physics & Astronomy, University of the Western Cape, Private Bag X17, Bellville, 7535 South Africa; 5 TRIUMF, Vancouver, V6T 2A3 BC Canada; 6 Physics Department, Lund University, Box 118, Lund, SE-221 00 Sweden; 7 Department of Physics, University of Michigan, Ann Arbor, 48104 MI USA; 8 INFN Laboratori Nazionali di Legnaro, Legnaro, 35020 PD Italy; 9 Instituut voor Kern- en Stralingsfysica, KU Leuven, Leuven, B-3001 Belgium; 10 Department of Physics, University of Guelph, Guelph, N1G 2W1 Ontario Canada; 11 Institute for Nuclear Physics, University of Cologne, Cologne, 50937 Germany; 12 Institut für Kernphysik, Technische Universität Darmstadt, Darmstadt, 64289 Germany; 13 Heavy Ion Laboratory, University of Warsaw, Warsaw, PL-02-093 Poland; 14 Department of Physics, University of Jyvaskyla, P.O. Box 35, Jyvaskyla, FIN-40014 Finland; 15 Helsinki Institute of Physics, P.O. Box 64, Helsinki, FIN-00014 Finland; 16 Department of Physics, University of Oslo, P.O. Box 1048, Oslo, N-0316 Norway; 17 Department of Physics, University of York, York, YO10 5DD UK; 18 JINR Dubna, Dubna, 141980 Moscow Region Russia; 19 Consejo Superior De Investigaciones Científicas, Madrid, S 28040 Spain; 20 IRFU CEA, Université Paris-Saclay, Gif-sur-Yvette, F-91191 France

**Keywords:** Exotic atoms and molecules, Experimental nuclear physics

Correction to: *Nature Communications* 10.1038/s41467-019-10494-5, published online 06 June 2019.

The original version of this Article contained an error in the third sentence of the ‘Data selection and Doppler correction’ section of the Methods, which incorrectly read “In the present setup the average angle of each of the 16 strips of one side of the silicon detector array ranged between 19.6° and 54.9° to the beam direction, corresponding to a scattering angular range of 140.9°–0.2° in the centre-of-mass”. The correct version states ‘140.9°–70.2°’ in place of ‘140.9°–0.2°’. This has been corrected in both the PDF and HTML versions of the article.

